# Translation and cross-cultural adaptation of the Nepali version of the Rowland universal dementia assessment scale (RUDAS)

**DOI:** 10.1186/s41687-019-0132-3

**Published:** 2019-07-19

**Authors:** Govinda Mani Nepal, Asmita Shrestha, Ranjeeta Acharya

**Affiliations:** 1 Department of Physiotherapy, Kathmandu University School of Medical Sciences, Dhulikhel, Nepal; 2 Nepal Medicity Hospital, Bhaisepati, Nepal

**Keywords:** Cognition, Mild cognitive impairment, Elderly, RUDAS, Nepali

## Abstract

**Background:**

Mild Cognitive Impairment (MCI) is a transitional state between normal cognition and dementia during ageing process. Early screening of elderly for MCI can help for early prevention and treatment of dementia. Rowland Universal Dementia Assessment Scale (RUDAS) is a brief cognitive assessment tool with excellent psychometric properties and is particularly useful in culturally and linguistically diverse (CALD) populations. The purpose of this study was to translate RUDAS into Nepali and to evaluate internal consistency of Nepali version of RUDAS.

**Methods:**

RUDAS was translated to Nepali following recommended guidelines for translation and cross-cultural adaptation of patient reported measures. The pre-final Nepali version of RUDAS was tested on 30 elderly and appropriate changes were made by expert committee. The final Nepali version of RUDAS was developed and was administered on 100 elderly populations (mean age 67.70) to assess internal consistency.

**Results:**

The rating of the participants upon their understanding of each 6 items of Nepali version of RUDAS on a 10-point Likert scale received a good score of more than 8 except ‘cube drawing’ which only received a mean score of 5.5. Additionally, only 8(27%) elderly have responded to the item ‘cube drawing’. The preference test was done and cube drawing was replaced by stick design test. Nepali version of RUDAS showed acceptable internal consistency with Cronbach’s alpha 0.7.

**Conclusion:**

The results of our study presents translated and cross-culturally adapted Nepali version of RUDAS that has been proved to be an appropriate assessment tool for screening cognitive impairment among elderly.

**Electronic supplementary material:**

The online version of this article (10.1186/s41687-019-0132-3) contains supplementary material, which is available to authorized users.

## Introduction

Cognitive impairment and dementia are global public health problems. Dementia is a chronic neurodegenerative disease in which there is deterioration of various aspects of cognition including memory, thinking, judgment, orientation, learning capacity, comprehension and language [1]. Mild Cognitive Impairment (MCI) is a transition phase between normal cognition and dementia and it affects any one of the aspects of cognitive function in elderly [1].

According to World Health Organization, worldwide, 47.5 million people have dementia and 58% of these are from low and middle income generating countries (LMICs) [2]. There are 7.7 million new cases of dementia every year and this number is projected to get doubled by 2030 and almost tripled by 2050 [2]. Similarly, the world population is also rapidly aging and population of elderly (60 years and above) is considered to be doubled by 2050 [3]. These elderly are at higher risk of developing neurodegenerative diseases like dementia and MCI. In Nepal, the population of people aged 60 years and above is 2.1 million (8%) [4]. They are at high risk of developing dementia in next few years [5, 6].

Cognitive impairment impacts on activities of daily living, financial issues and quality of life which increases the psychological morbidity, social isolation, physical ill-health and poor immunity as well as burden on family members [7]. Earlier identification of cognitive impairment or dementia reduces patients and caregivers morbidity and also helps to identify risk of complications [7]. However, health care professionals often fail to recognize cognitive impairment in earlier stages and the prevalence of missed diagnosis has been found to be 25%–90% [8].

Thus, a reliable and valid screening tool is necessary to assess cognitive function of patient [7]. There is a wide range of cognitive assessment tools and about 40 different types of tests are available for cognitive screening [9]. The Rowland Universal Dementia Assessment Scale (RUDAS) is a reliable and valid cognitive assessment tool that was created for culturally and linguistically diverse (CALD) populations [10]. RUDAS is a 6-item interview based questionnaire that assesses multiple domains of cognitive functions namely; ‘memory recall’, ‘visuospatial orientation’, ‘praxis’, ‘visuoconstructional drawing’, ‘judgment’ and ‘language’. A culturally relevant and comprehensible form of a tool is very important to use in clinical practice and research. Thus, the objective of this study was to translate and cross culturally adapt RUDAS in Nepali language.

## Methods

### Study design and settings

The translation and cross cultural adaptation of RUDAS was conducted at Kathmandu University School of Medical Sciences, Kavre, Nepal. Translation was carried out following a standardized guideline [11, 12]. A cross-sectional study was conducted to evaluate the internal consistency of RUDAS.

The ethical approval was obtained from Institutional Review Committee of Kathmandu University School of Medical Sciences, Dhulikhel, Nepal (reference number: 52/17). Written consent was obtained from all the participants prior to data collection.

### Translation of RUDAS into Nepali language

**Fig. 1:**
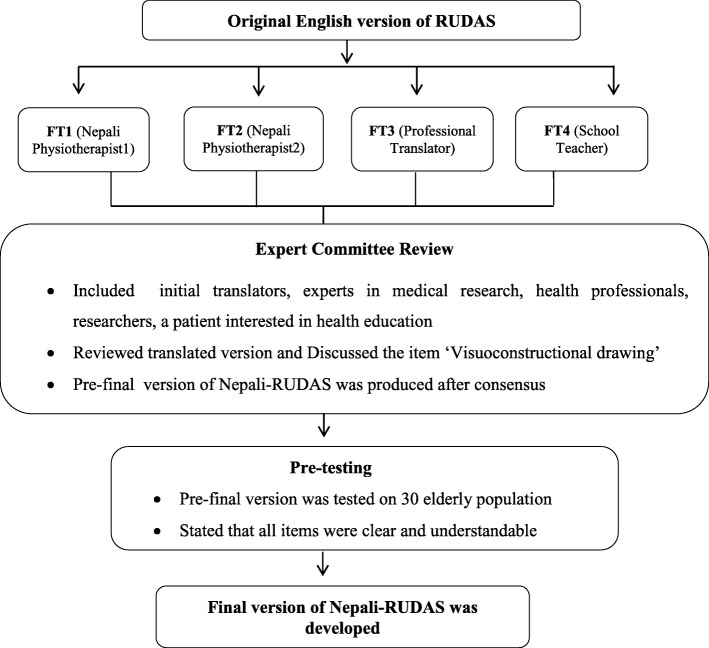
Process of translation and cross-cultural adaptation of RUDAS

We obtained prior permission from the original developer of RUDAS. The translation and cross cultural adaptation process was conducted using “Guidelines for the process of cross-cultural adaptation of self-reported measures” as recommended by Beaton et al. [11] However, the back translation was not performed as suggested by Epstein et al. [12] since it does not have added benefit compared with expert committee review only and that it can be avoided where committee is proficient enough [13]. The process included following four stages (as shown in Fig. 1) 1) Forward translation 2) Synthesis 3) Expert committee review 4) Pretest of the measure. After the translation, internal consistency of RUDAS was calculated among 100 elderly.

The translation process included following steps:

- **Forward translation (FT):**

Four translators- two from medical background (FT1 and FT2: working as a physiotherapist) and two others from non-medical background (FT3: professional translator and FT4: school teacher) were chosen to independently translate the English version of RUDAS into Nepali. These translators were bilingual with their mother tongue being Nepali language. FT1 and FT2 were aware of the concepts being examined in the questionnaire, while FT3 and FT4 were unaware and were not informed of the concepts in the questionnaire.

- **Synthesis of FT1, FT2, FT3 and FT4 (a common Nepali translation):**

A consensus meeting between the researchers, with the original version of the questionnaire was held and a common (FT-1234) synthesis was produced with a written report carefully documenting the synthesis process. During the synthesis process, appropriate Nepali words for various terms were discussed and a pre-final version was synthesized.

- **Expert committee review:**

The expert committee comprised of group of experts that included the initial translators, experts in medical research, health professionals, one patient interested in health education and researchers involved in studies of translation. Expert committee sorted out discrepancy between source and target version in all four areas; semantic equivalence, idiomatic equivalence, experimental equivalence and conceptual equivalence [11]. The words that were difficult to translate were also reviewed. After the consensus from the experts, researchers and the translators, pre-final version of the questionnaire was prepared.

- **Testing of pre-final version of the questionnaire:**

Pre-final Nepali version of RUDAS was tested on 30 individuals aged 60 years and above. The participants were asked to complete the questionnaires and were additionally interviewed with open questions to find the differences between the meaning of the items and their actual responses. They were asked to rate their understanding of all the 6 items of Nepali version of RUDAS on a 10-point numeric rating scale (where 0 is not at all understandable and 10 is completely understandable). The responses on all the items were further discussed in the expert committee along with proportion of missing responses. We noticed that there was 100% response in all the items except for ‘visuoconstructional drawing’ where only 8(27%) elderly had responded. These participants responded that they were unaware of the cube used for ‘visuoconstructional drawing’ and did not know how to draw the cube. To observe the ability and preference of Nepalese elderly, the expert committee decided to use three different tests of visuoconstructional ability: ‘Stick design, clock drawing and cube drawing’ on 26 elderly. Based on the results of the preference testing, final version of translated Nepali RUDAS was produced (see Additional file 1 for translated Nepali RUDAS).

### Internal consistency of RUDAS

A total of 104 elderly were screened for internal consistency of RUDAS. Participants with presence of any speech abnormalities (aphasia, dysarthria, dysphonia) and/or unable to understand Nepali language were excluded from the study. Among 104 elderly, three refused to complete the questionnaire and one of them was unable to complete due to dyspnea.

Informed consent was obtained from all the individuals before the administration of questionnaire. Initially, demographic details were asked including name, age, sex, address, education and marital status. Then, Nepali -RUDAS was administered.

### Data analysis

Statistical analysis was done by using software, Statistical Package for Social Sciences version (SPSS) 16. The baseline characteristics of study population were analyzed using descriptive statistics. Cronbach’s alpha was used to compute internal consistency of the responses.

## Results

A) Forward translation, synthesis and expert committee review

Linguistic differences were noted between forward translators regarding language of use in several items. In the expert committee meeting, there were discussions on all the items of RUDAS regarding literal meaning and proper grammar. Out of them, four items namely, ‘visuospatial orientation’ (item 2), ‘visuoconstructional drawing’ (item 4), ‘memory recall’ and ‘recall’ (item 1) were difficult to translate. Appropriate Nepali word or phrase was difficult to identify, as literal translation was not applicable. However, after the consensus between the translators and others experts in the meeting, *‘drishya-duri (visuospatial) abhimukhikaran’, ‘drishyanirmandchitran’, ‘puna-smaran’* and *‘aba samjhanuhos’* were used for ‘visuospatial orientation’, ‘visuoconstructional drawing’, ‘memory recall’ and ‘recall’ respectively.

Significant discussion was also done regarding the translation of phrase: ‘Demonstrate at moderate walking pace’ of item ‘Praxis’ (item 3). Translators were more focused on ‘walking’ part and phrases like *‘manda hidaiko gatima dekhaune’* which meant *‘demonstrate walking at moderate pace’, ‘thikka hidne gatima dekhaunuhos’* which meant *‘ demonstrate precise walking pace’* etc. were suggested. None of these phrases sounded natural and during expert committee review, it was concluded that the phrase meant demonstration at a moderate pace not walking. Thus, it was translated as *‘madhyam gatima pradharsan garne’* which simply means *‘demonstrate at moderate pace’.* Discussions were also made regarding proper grammar, conciseness and clarity of all other items.

The agreement was also made for the rating scale of item 3 (praxis) where inconsistency was observed among the translators and the pre-final version of questionnaire was developed with all the necessary changes.

B) Testing of the pre-final version of the questionnaire

The pre final version of Nepali-RUDAS was tested on 30 elderly. The participants were 10(33%) male and 20 (67%) female with mean age of 69.63 ± 6.91. Majority of participants were uneducated 19 (63%). Table 1 demonstrates the baseline characteristics of the participants.

**Table 1:** Demographic details of participants in testing pre-final version of Nepali-RUDAS

Variable	N(%) or mean SD
Age	69.63 ± 6.911
Gender, N (%)	
Male	10 (33%)
Female	20 (67%)
Educational status, N (%)	
Uneducated	19 (63%)
Primary level	3 (10%)
Secondary level	1 (3%)
High school / Certificate level	7 (23%)
Bachelors level	0 (0%)

The participants were asked to complete the questionnaires and helping them understand the questionnaire if required. The participants rated their understanding of each 6 items of Nepali version of RUDAS on a 10-point Likert scale. All the items received a mean score of more than 8 except ‘Visuoconstructional drawing’ which received only a mean score of 5.5 (Table 2). We also noticed that there was 100% response in all the items except for an item ‘visuoconstructional drawing’ where only 8(27%) elderly have responded to that item. The remaining 22(73%) left it unanswered. The participants were further interviewed on why they left this item unanswered and gave less score if answered at all. The majority of participants responded ‘maile padeko chaina ra malai yesto banauna aaundaina’ which means ‘I am not educated person and I do not know to draw this’. Others responded ‘maile yesto dekheko chaina ra banauna aaundaina’ which means ‘I am completely unaware of the cube used for drawing and do not know to draw the cube’. We further conducted the correlation analysis between the education level of the participants and their ability to understand the cube drawing. There were significant correlations between participants educational level and their ability to understand cube drawing(*r* = 0.90, *P* = 0.001). To observe the ability and preference of Nepalese elderly, the expert committee further decided to use three different tests of visuoconstructional ability: ‘Stick design, clock drawing and cube drawing’. The preference testing was done on 26 elderly for an item ‘visuoconstructional drawing’. These were the separate participants from the original sample. The mean age of participants was 66.96 ± 6.914.26 with 62% (*n* = 16) male and 38% (*n* = 10) female (Table 3). The total test failure of stick design test, cube drawing and clock drawing was 0%, 85% and 62% respectively as shown in Table 3. It shows that stick design test was more acceptable than other tests. Out of the three tests used, stick design test was the most preferred one (89%, *n* = 23) followed by clock drawing (8%, n = 2) and cube drawing (4%, n = 1) as shown in Table 3. The results showed that the stick design test was more acceptable than other tests and thus ‘cube drawing’ of item ‘visuoconstructional ability’ was replaced by stick design test (See Additional file 2 for more information on stick design test).

**Table 2:** Likert scale (understanding the items of Nepali-RUDAS)

Items	Response (N)	Minimum	Maximum	Mean	SD
Visuospatial orientation	30	7	10	8.93	1.143
Praxis	30	6	10	8.47	1.332
Visuoconstructional drawing	8	4	7	5.50	1.195
Judgement	30	6	10	8.40	1.248
Memory recall	30	7	10	8.90	.995
Language	30	7	10	8.97	1.066

**Table 3:** Results of preference testing

Demographic details N (%)	Tests	Frequency of test failure (%)	Preference
Male −16 (61.5%)	Stick design test	0 (0%)	89%
Female −10 (38.5%)	Cube drawing	22 (84.6%)	4%
Mean age- 66.96 ± 6.914	Clock drawing	16 (61.5%)	8%

Apart from the above mentioned items, other items were also discussed. The ‘cooking oil’ of the item ‘Memory recall’ (item 1) was responded as just ‘oil’ by 60% elderly. Considering the essence of the item, expert committee decided to keep the same Nepali word “*pakaune tel”* for cooking oil. Eleven participants (36.6%) reported that it was difficult for them to understand a term ‘Traffic lights’ of an item judgment (item 5). Most of them asked what ‘traffic’ means and few misunderstood it as ‘traffic light stick’. To address their misinterpretation, experts decided on using an additional word “*Sadak batti (Traffic batti)*” which means ‘Road lights’ to make it understandable.

C) Internal consistency of RUDAS

100 elderly were included in the study with 54% (*n* = 54) male and 46% (*n* = 46) female participants. Mean age of the participants was 67.72 ± 6.08 and the mean RUDAS score was found to be 25.59 ± 4.17 as shown in Table 4. Only 16% of the sample had cognitive impairment at cut off score of 22. The Internal consistency was found to be acceptable with Cronbach’s alpha equal to 0.7. There were non-significant correlations of RUDAS scores with participant’s age (*r* = − 0.11, *P* = 0.273) and educational level (*r* = + 0.22, *P* = 0.022).

**Table 4:** Demographic details and RUDAS scoring in computing internal consistency

Variable	N(%) or mean SD
Ages	67.72 ± 6.084
RUDAS score	25.59 ± 4.176
Gender, N (%)	
Male	54 (54%)
Female	46 (46%)
Marital Status, N (%)	
Married	75 (75%)
Unmarried	1 (1%)
Widow	24 (24%)
Educational status, N (%)	
Uneducated	60 (60%)
Primary level	15 (15%)
Secondary level	8 (8%)
High school / Certificate level	3 (3%)
Bachelors level	6 (6%)
Informal education	8 (8%)

## Discussion

RUDAS was translated and cross culturally adapted into Nepali language following the recommended translation guidelines [11, 12]. Originally, RUDAS was designed to enable easy translation and is recommended by Expert Clinical Reference Group (ECRG) for use in people with CALD background like Nepal [14, 15]. It has also been found that RUDAS correlates well with MMSE, was less time consuming and had similar patient satisfaction [16]. The results of RUDAS were less affected by language and education level than MMSE [17]. RUDAS has been successfully translated and validated in various countries, without the need of any changes in the content [18–21]. However, in our study, the translation was accomplished with minor adaptations made relevant to Nepali community.

We found language differences in several items during the forward translations and synthesis stage. It correlates with the fact that in Nepal, there are about 125 castes identified and around 123 different languages are spoken throughout the country [15]. The differences in the languages were discussed during the expert committee meeting and resolved by bringing consensus.

The forward translation and synthesized version demonstrated different language for the item “Demonstrate at moderate walking pace” of item ‘Praxis’. We found that the translators were focused more with ‘walking’ rather than ‘moderate’ part. Hence the committee decided to make it more precise and translated it as *“madhyam gatima pradharsan garne”.* Most of the items in RUDAS were well understood by the participants similar to various translation and validation studies of RUDAS where they reported no changes in its content during translation process [18–21]. This was likely because RUDAS was originally developed for culturally diverse populations and it consists of universal items without superimposing language of any particular background. However, there were exceptions in our study for the items ‘visuoconstructional drawing’ and ‘Judgement’. The inability to understand “traffic lights” of the item ‘judgement’ was possibly because Nepalese are unaccustomed of the word ‘traffic’. The committee thus decided to translate it as “*Sadak batti(Traffic batti)”* with an additional hint provided in a bracket which means “road lights” in English. Similarly, to address inability of the participants to draw a cube, the expert committee discussed and decided to perform preference testing for ‘visuoconstructional drawing’ item using different tests (‘Stick design, clock drawing and cube drawing’) [22–25]. Based on the participants comments, the stick test was most understandable test which was then decided to be modified from the original ‘visuoconstructional drawing’ item where cube drawing is used. This is in contrast to the findings of the validation study by Storey et al. where they reported that RUDAS can be translated into other languages without the need to change the structure or the format of any items [10].

We found strong correlation between participant’s educational level and their ability to understand the cube drawings in “visuoconstructional drawing” item. Majority of our study participants were uneducated (63%).Conflicting evidence about the effect of education on RUDAS scoring were observed as some studies [10, 26, 27] reported positive influence of educational level and others [18, 20, 21] showing no influence at all. This could possibly because of different cultural background and preference of language in different countries. However, the findings of our study is supported by a study from Chaaya et al. where significant numbers of participants were unable to perform cube drawing test because of their low educational status [18]. Additionally, the cube drawing test when administered on less educated persons showed negative emotional reactions and very low scores [24, 25]. Our results is also similar to the study done in Nigeria, which showed that the stick design test was more acceptable than cube drawing [22]. Likewise, in a validation study of Arabic version of RUDAS, test failure for cube drawing was 51%, 30% and 14% for participants with no formal education, primary to secondary education and university education respectively [18].

We found Internal consistency of Nepali-RUDAS was acceptable. It signifies that all the items of RUDAS measures the common attribute i.e. cognition. The cross-sectional study performed on 130 participants in China by C-W Chen et al. found internal consistency to be 0.71 which is similar to our study [19]. Our study supports the fact that RUDAS is a useful screening test to detect cognitive impairment in Nepalese clinical settings.

### Strengths and limitations

The strengths of this study are the sample from different communities, applicability to both gender as our participants inclusion were both male and female, and inclusion of participants from different educational background. We used standardized guidelines for translation and adaptation [11] without back translation as suggested by Epstein et al. [12] Internal consistency of RUDAS was assessed in 100 participants which is considered adequate as per the COSMIN guidelines [28]. We were unable to screen the participants with any cognitive impairment due to lack of valid instrument available in Nepali which can be considered as limitations of the study.

### Recommendations

It would be advisable to conduct further validation studies of Nepali-RUDAS in different settings and in larger groups. We strongly recommend assessing test-retest reliability, validity and responsiveness of N-RUDAS in near future. Furthermore, RUDAS was primarily designed to detect dementia for elderly populations. Thus, more researches are recommended for its application in younger age groups as well as other neurological conditions. The internal consistency of N-RUDAS was 0.70 which is considered just adequate and we recommend conducting further researches to identify more reliable and valid tool to assess cognitive function in Nepali population.

## Conclusion

The Nepali version of RUDAS (Nepali-RUDAS) is a reliable tool for the screening of cognitive impairment among elderly. It is comprehensive, culturally appropriate and user friendly instrument for use in clinical settings as well as research purposes. The availability of this measure will encourage and facilitate decision making and further researches.

## Additional files

Additional file 1: N- RUDAS. (DOCX 742 kb)

Additional file 2: Stick Design Test. (DOCX 18 kb)

## Data Availability

All data generated or analyzed during this study are included in this article.

## Abbreviations

CALD: Culturally And Linguistically Diverse
COSMIN: Consensus-Based Standards For The Selection Of Health Measurement Instruments
ECRG: Expert Clinical Reference Group
LMICs: Low and Middle Income generating Countries
MCI: Mild Cognitive Impairment
MMSE: Mini Mental State Examination
RUDAS: Rowland Universal Dementia Assessment Scale
SD: Standard deviation
SPSS: Statistical Package for Social Sciences
