# Supplementary figures and images for: Translation and cross-cultural adaptation of the Nepali version of the Rowland universal dementia assessment scale (RUDAS)
Source: J Patient Rep Outcomes. 2019 Jul 19;3:38. doi: 10.1186/s41687-019-0132-3 (PMC6639471; doi:10.1186/s41687-019-0132-3)

**Appendix 1: N- RUDAS**


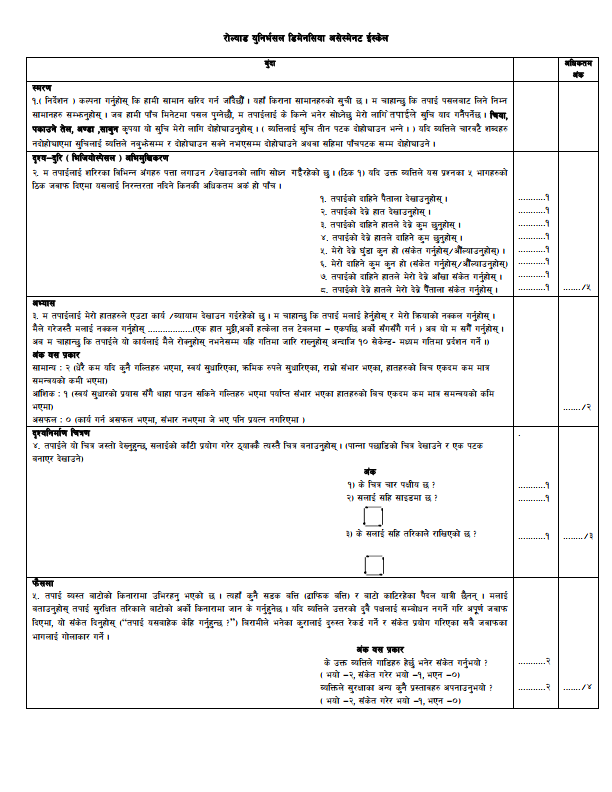


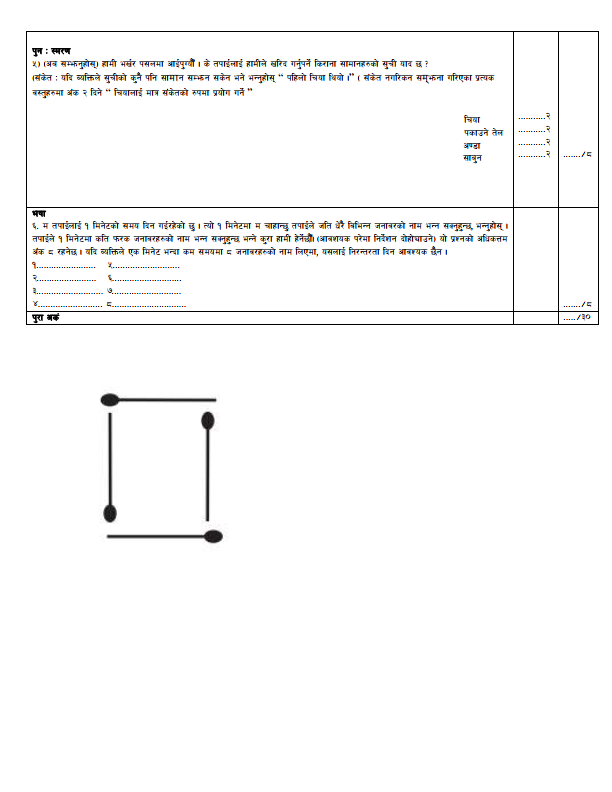

Supplement: Supplementary file 1 — N- RUDAS. (DOCX 742 kb) [file 41687_2019_132_MOESM1_ESM.docx]
