# Supplementary material for: Translation and cross-cultural adaptation of the Nepali version of the Rowland universal dementia assessment scale (RUDAS)
Source: J Patient Rep Outcomes. 2019 Jul 19;3:38. doi: 10.1186/s41687-019-0132-3 (PMC6639471; doi:10.1186/s41687-019-0132-3)

**Appendix 2: Stick Design Test**

In Stick design test, a representation of an arrangement of four wooden matches is printed on a paper. The designs approximate a square, a
triangle with stem, a chevron, and a rake-like figure. In our context, last three items were less relevant thus square was chosen for pilot testing.

Procedure: The examiner demonstrates how to arrange the matches to copy the stimulus, explicitly noting in the process the need to correctly orient the match heads. The match heads are then collected and handed to the participant who is told to make an exact copy of the stimulus.

Scoring:

Is it a four sided figure? (Yes: 1, No: 0)

Does the figure rest on a side? (Yes: 1, No: 0)

Are match heads correctly oriented? (Yes: 1, No: 0)


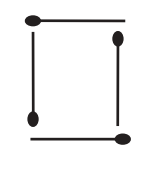

Supplement: Supplementary file 2 — Stick Design Test. (DOCX 18 kb) [file 41687_2019_132_MOESM2_ESM.docx]
